# STXBP3 and GOT2 predict immunological activity in acute allograft rejection

**DOI:** 10.3389/fimmu.2022.1025681

**Published:** 2022-12-01

**Authors:** Qinfan Yao, Cuili Wang, Yucheng Wang, Wenyu Xiang, Yin Chen, Qin Zhou, Jianghua Chen, Hong Jiang, Dajin Chen

**Affiliations:** ^1^ Kidney Disease Center, The First Affiliated Hospital, College of Medicine, Zhejiang University, Hangzhou, China; ^2^ Key Laboratory of Kidney Disease Prevention and Control Technology, Hangzhou, China; ^3^ Institute of Nephropathy, Zhejiang University, Hangzhou, China; ^4^ Zhejiang Clinical Research Center of Kidney and Urinary System Disease, Hangzhou, China

**Keywords:** acute rejection, kidney transplantation, expression, STXBP3, GOT2

## Abstract

**Background:**

Acute allograft rejection (AR) following renal transplantation contributes to chronic rejection and allograft dysfunction. The current diagnosis of AR remains dependent on renal allograft biopsy which cannot immediately detect renal allograft injury in the presence of AR. In this study, sensitive biomarkers for AR diagnosis were investigated and developed to protect renal function.

**Methods:**

We analyzed pre- and postoperative data from five databases combined with our own data to identify the key differently expressed genes (DEGs). Furthermore, we performed a bioinformatics analysis to determine the immune characteristics of DEGs. The expression of key DEGs was further confirmed using the real-time quantitative PCR (RT-qPCR), enzyme-linked immunosorbent assay (ELISA), and immunohistochemical (IHC) staining in patients with AR. ROC curves analysis was used to estimate the performance of key DEGs in the early diagnosis of AR.

**Results:**

We identified glutamic-oxaloacetic transaminase 2 (GOT2) and syntaxin binding protein 3 (STXBP3) as key DEGs. The higher expression of STXBP3 and GOT2 in patients with AR was confirmed using RT-qPCR, ELISA, and IHC staining. ROC curve analysis also showed favorable values of STXBP3 and GOT2 for the diagnosis of early stage AR.

**Conclusions:**

STXBP3 and GOT2 could reflect the immunological status of patients with AR and have strong potential for the diagnosis of early-stage AR.

1 Introduction

Chronic kidney disease (CKD) (1–3) is a worldwide public health issue and constitutes an increasing economic burden owing to its high morbidity. According to the United States Renal Data System (USRDS 2021), 130,400 people were newly diagnosed with end-stage renal disease (ESRD) in 2019, an increase of 2.5% over the previous year. Kidney transplantation (KT) (4–8) is considered the optimal choice for treating ESRD. The recent introduction of novel immunosuppressive agents has reduced the occurrence of one-year acute rejection (AR) following kidney transplantation (9–12). Nevertheless, early acute rejection still affects approximately 10– 15% (13–15) of recipients after the first post-kidney transplant year. According to the 2019 OPTN/SRTR Annual Kidney Data Report, 7.0% of kidney transplant recipients suffer from AR within 1 year after transplantation, comprising 9.1% of recipients aged between 18 and 34 years and 6.1% of recipients aged>65 years. More importantly, AR is a crucial factor affecting the long-term function and survival of transplanted kidneys. Graft survival and patients’ long-term outcomes deteriorate once AR has occurred (16–18). AR is typically diagnosed by increased serum creatinine concentration (Scr), decreased glomerular filtration rate (GFR), changes in urine volume, and biopsy (19, 20). Unfortunately, these indicators are delayed and insensitive biomarkers of AR (21–24), usually reflecting a late sign of kidney damage. Therefore, the identification of more sensitive and specific biomarkers for the early diagnosis of acute renal rejection is imperative (19).

In recent years, RNA sequencing (RNA-Seq) using next-generation sequencing (NGS), a valuable alternative to microarrays, has been widely applied. An increasing number of novel plasma and urinary biomarkers have been explored for monitoring AR (25–27), including neutrophil gelatinase-associated lipocalin (NGAL), donor-derived cell-free DNA (dd-cfDNA) (28, 29), CXCL9 and CXCL10 (30–32). However, there are still no effective biomarkers that can accurately diagnose early stages of AR (29, 31, 33–35).

In this study, RNA-seq analysis of peripheral blood samples obtained before and after surgery was used to detect the dynamic changes in the pre- and postoperative DEGs. The combination of five online gene expression microarray datasets and our center gene expression mRNA-Seq analysis was utilized to filter out pre- and postoperative DEGs. Bioinformatics analysis and the xCell website were used to further screen the genes associated with immune response and map these selected genes to the corresponding immune cells. Then, the expression of selected genes at transcriptional and translational levels was determined using real-time quantitative real-time PCR (RT-qPCR), enzyme-linked immunosorbent assay (ELISA), and immunohistochemical (IHC) staining. Two promising genes were identified, namelySTXBP3 and GOT2, through continuous screening of the key gene set. Subsequently, ROC curve analysis was applied to validate the AR early diagnostic value of STXBP3 and GOT2 in the serum of patients before undergoing kidney transplantation. We demonstrated that STXBP3 and GOT2 are sensitive biomarkers for early AR diagnosis.

2 Materials and methods

2.1 Data collection

The study recruited 42 patients who underwent on renal transplantation between 2018-01-01 and 2019-01-31 due to ESRD in the Kidney Disease Center of the First Affiliated Hospital, College of Medicine, Zhejiang University, which included 23 patients with AR episodes and 19 patients without AR episodes (NAR). The inclusion criteria were ESRD patients aged>18 years who had received conventional treatment for at least 3 months. Exclusion criteria included acute kidney injury, active inflammatory or malignant disease, pregnancy, and inability to provide informed consent. Kidney biopsies were performed for all patients. The Banff criteria were used to define acute rejection based on kidney biopsy data. Peripheral blood mononuclear cells (PBMCs) from allograft recipients were stored at -80°C until used. Informed consent was obtained from all patients enrolled in this study.

2.2 RNA sequencing

TRIzol reagent (Invitrogen, Carlsbad CA, US) was used to obtain the total RNA (1000 ng) of PBMCs. The concentration and purity of the extracted RNA were determined using a NanoDrop2000 spectrophotometer (Thermo Fisher Scientific, Waltham, MA, USA). RNA integrity was confirmed using the 2100 Bioanalyzer (Agilent, Santa Clara, CA, USA). Samples with an RNA integrity number (RIN) > 8 were screened for RNA sequencing. mRNA-sequencing was performed using Illumina HiSeq X-ten according to the manufacturers' protocols. The ComBat R package (https://intro2r.com/citing-r.html) was used to conduct log2 conversion, quantile normalization, and experimental batch correction on the read counts in comparison with the transcription levels among the samples (36). The fragments per kilobase transcriptome per million mapped reads (FPKM) of each gene were computed according to the gene length and mapped read count. DEG analysis was performed to identify patients with AR and NAR using the LIMMA R package (37). Genes with a p < 0.05 and log2 (fold change, FC) ≥ 1.5 were considered significant. To correct for multiple comparisons for screening thDEGs, FDR was applied to p< 0.05, which was considered significant.

2.3 Identification of DEGs involved in acute rejection

The study design is illustrated in **Figure 1**. DEGs related to AR in kidney transplantation were identified, by obtaining five gene expression microarray data sets from the NCBI Gene Expression Omnibus database (www.ncbi.nlm.nih.gov/geo/) with accession numbers GSE112927, GSE120396, GSE120649, GSE131179 and GSE145503. Additionally, we included mRNA-Seq-based gene expression data from our cohort of 42 patients in this study. The six data sets included two pretransplant (GSE112927, our center set) and four post-transplant (GSE120396, GSE120649, GSE131179 and GSE145503) datasets.

**Figure 1:**
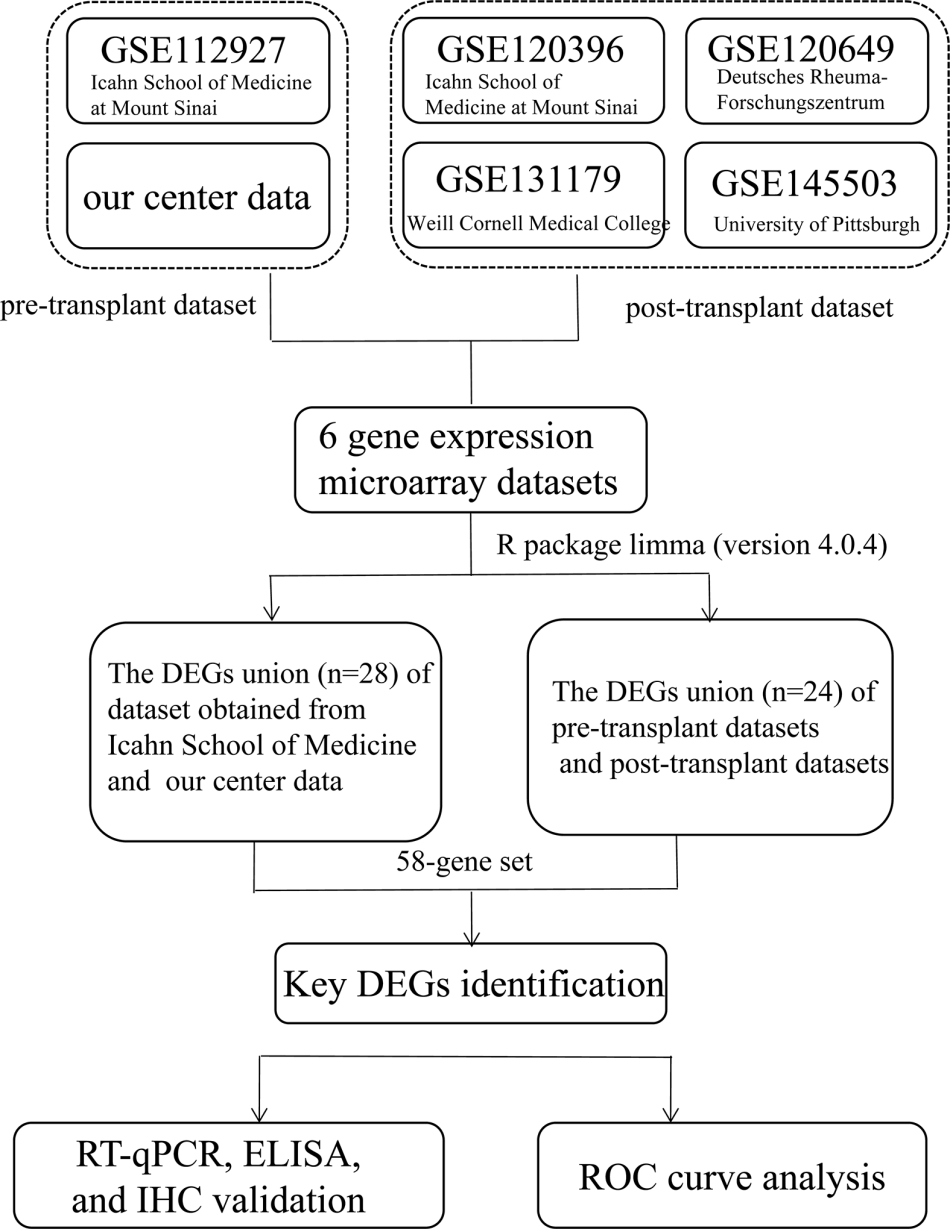
Flowchart depicting the overall study design. A 58-gene dataset was aligned with those expressed by 64 immune cells in xCell and the key genes were identified. Fifteen key DEGs were identified for further validation (KIF3B, FH, EIF4G1, SMARCD1, ITGAL, HNRNPUL1, MAP4K5, ELP3, AVIL, HNRNPL, PRPF19, GOT2, STXBP3, CLIC3, and PPM1G).

The six datasets included in this study were generated using different sequencing platforms such as Illumina HiSeq - 4000, 2000, 2500, 2500 and NextSeq 500. DEG analysis was performed using LIMMA R package (37).

Venn diagrams (http://bioinformatics.psb.ugent.be/webtools/Venn/) were used to represent the intersecting DEGs among the six datasets (38) (**Figure 1**). To further explore the correlation between genes and immune cells, we subsequently filtered genes by the condition in which they were expressed by 64 types of immune cells on the xCell website (https://xcell.ucsf.edu/) (39). Finally,15 key genes were identified for further analysis.

BioJupies was used to generate heatmap, and perform enrichment analysis of STXBP3, GOT2, and MAP4K5. Statistical significance was set at p < 0.05 (40).

2.4 DEG validation using RT-qPCR, ELISA, and IHC staining

The expression of 15 DEGs (kinesin family member 3B (KIF3B); fumarate hydratase (FH); eukaryotic translation initiation factor 4 gamma 1 (EIF4G1); SWI/SNF related, matrix associated, actin dependent regulator of chromatin, subfamily d, member 1 (SMARCD1); integrin subunit alpha L (ITGAL); heterogeneous nuclear ribonucleoprotein U like 1 (HNRNPUL1); mitogen-activated protein kinase kinase kinase kinase 5 (MAP4K5); elongator acetyltransferase complex subunit 3 (ELP3); advillin (AVIL); heterogeneous nuclear ribonucleoprotein L (HNRNPL); pre-mRNA processing factor 19 (PRPF19); glutamic-oxaloacetic transaminase 2 (GOT2); syntaxin binding protein 3 (STXBP3); chloride intracellular channel 3 (CLIC3); and protein phosphatase, Mg2+/Mn2+ dependent 1G (PPM1G)) were found to significant when RT-qPCR was performed. RNA was first extracted from each sample using QIAamp MinElute Virus Spin kits followed by quantification using a NanoDrop2000 spectrophotometer and BioAnalyzer. cDNA was synthesized from 10 μL of RNA using the PrimeScriptTM RT reagent kit (TaKaRa, Shiga, Japan). The subsequent RT–qPCR screening of the enrolled subjects for the 15 markers was performed using the primers listed in **Supplementary Table 1**. Gene-specific primers used for RT-qPCR were designed using PrimerBank. GAPDH was used as an internal control to normalize the relative levels of mRNA and the 2−ΔΔCT method was used to analyze the fold change in mRNA expression. Subsequently, ELISA was performed to detect the levels of STXBP3 (abx383548, Abbexxa, Texas, USA), GOT2 (OKEH04266, Aviva Systems Biology, San Diego, USA), and MAP4K5 (abx388413, Abbexxa, Texas, USA). The diluted samples were incubated in 96-well plates. After washing the membranes in TBS, they were incubated with diluted anti-horseradish peroxidase (HRP)-labeled antibodies at 37°C for 90mins. The absorbance was measured at 450 nm using ELISA microplate reader, and the corresponding concentrations were calculated using standard curve. Immunohistochemistry (IHC) was performed to measure the protein levels of STXBP3, and GOT2 following standard immunohistochemical technique. Sections (4 μm) were cut from the paraffin-embedded tissue block and pretreated with EDTA at 98°C for 30 min for antigen retrieval. The antibodies used were anti-STXBP3 (dilution 1:100; PA5-55549; Invitrogen) and anti-GOT2 (dilution 1:1000; MA5-36188; Invitrogen). Immunohistochemical evaluation was performed independently by two experienced pathologists who were blinded to the clinicopathological data.

2.5 Statistical analysis

Statistical analyses were performed using R version 4.0.4, SPSS version 26 (IBM), and GraphPad Prism 8.4.2. The AR diagnostic values for STXBP3, GOT2, and MAP4K5 were assessed using area under the ROC curve (AUC) analysis. Statistical significance was set at p < 0.05.

3 Results

3.1 Identification of key DEGs

Adetailed flow chart of the selection process for key DEGs is shown in **Figure 1**. First, the Venn diagram was used to obtain the intersection of GSE112927 and GSE120396 and to acquire the first group of 198 co-expressed genes (**Figure 2**). The same method was used to obtain 357 co-expressed genes at the intersection of our center and GSE120649, 758 co-expressed genes in intersection of our center and GSE131179, and 257 co-expressed genes at the intersection of our center and GSE145503. Next, the three groups of co-expressed genes were intersected with the first group of 198 co-expressed genes, the data of which were all conducted by Icahn School of Medicine at Mount Sinai, and three novel groups of co-expressed genes were obtained. Finally, the new three groups of co-expressed genes were merged and a total of 28 co-expressed genes (FH, TKT, SMARCD1, CLIC3, XRCC6, KHSRP, PPP1R3B, TOE1, ZFAND6, ITGAL, KIF3B, EIF4G1, FGFRL1, PRPF19, CD58, NABP1, PPM1G, TBCD, CCDC92, MDH2, PDIA4, SMARCAL1, GOT2, DNAJC11, MED24, BCL6, IFRD1, and STXBP3) were identified. Second,, the Intersection of two pretransplant datasets (GSE112927 and our center set) conducted using the Venn diagram obtained 2566 co-expressed genes. The intersection of four after transplantation datasets (GSE120396, GSE120649, GSE131179 and GSE145503) obtained using the Venn diagram revealed 162 co-expressed genes. Subsequently, the union of the two groups of co-expressed genes resulted in 24 co-expressed genes (TKT, MED27, AP2B1, BCL6, ZFAND6, GPR155, FH, NFE2L3, ITGAL, SMARCD1, MEF2A, CFH, PPP1R3B, AVIL, HNRNPUL1, ELP3, MAP4K5, KIF3B, EIF4G1, ZNF526, HSRP, FTSJ1, TOE1, and HNRNPL). Finally, the two sets of co-expressed genes obtained using the two steps above were merged to form a 58-gene dataset. Therefore, the xCell online tool was used to estimate the differentially infiltrated immune cells in the AR and NAR groups and found stronger infiltration of CD4+ T, CD8+ T, and plasma cells in the AR group. Genes were selected based on the immune features of the AR group that were expressed in the infiltrated immune cells in this group. Of the above-mentioned 58 genes, 15 expressed in differentially infiltrating immune cells were selected for further analysis (**Figure 3**).

**Figure 2:**
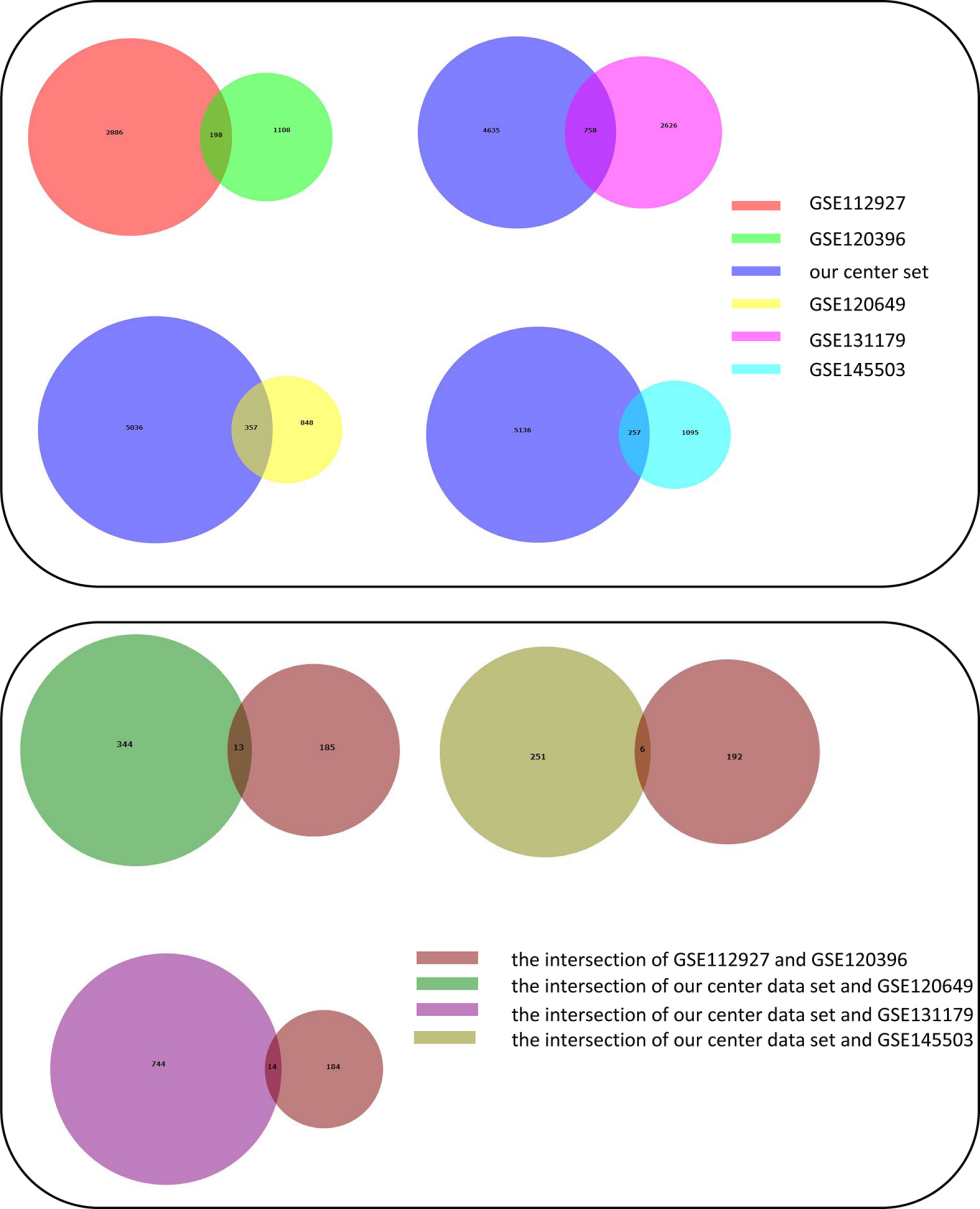
Construction of Venn diagrams for key DEGs identification in AR and NAR group. First method for identifying 28 co-expressed genes. The intersection of GSE112927 and GSE120396 yielded the first group of 198 co-expressed genes. The 357 co-expressed genes at the intersection of our center and GSE120649, 758 co-expressed genes at the intersection of our center and GSE131179, and 257 co-expressed genes at the intersection of our center and GSE145503 were obtained. The following three groups of genes were then intersected with the first group of 198 co-expressed genes. Three groups of co-expressed genes were merged to obtain 28 co-expressed genes (FH, TKT, SMARCD1, CLIC3, XRCC6, KHSRP, PPP1R3B, TOE1, ZFAND6, ITGAL, KIF3B, EIF4G1, FGFRL1, PRPF19, CD58, NABP1, PPM1G, TBCD, CCDC92, MDH2, PDIA4, SMARCAL1, GOT2, DNAJC11, MED24, BCL6, IFRD1, and STXBP3).

**Figure 3:**
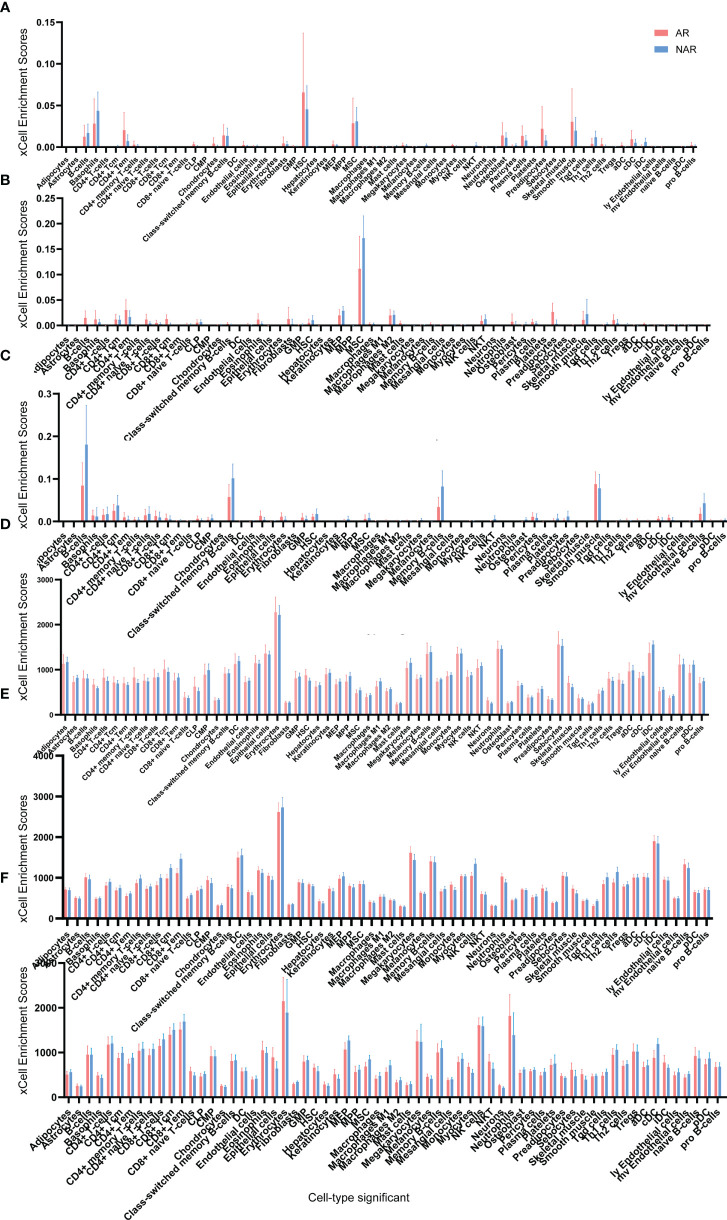
Cell type enrichment analysis of six RNA sequencing datasets. **(A)** GSE145503, **(B)** GSE131179, **(C)** GSE120649, **(D)** GSE120396, **(E)** GSE112927, and **(F)** our center) were performed using the xCell website. The x-axis lists the 64 cell types, and the y-axis depicts the xCell enrichment score (FDR < 0.1) in the AR group compared to the NAR group.

3.2 Clinical and demographic characteristics of patients with AR

Primary patient demographics and clinical features are shown in **Table 1**. In contrast to NAR recipients, the duration of dialysis was increased (*p*=0.042) and GFR was decreased (*p*=0.019) in the AR group. No statistically significant differences in other clinical indicators were observed between the two groups.

**Table 1:** Demographic and clinical characteristics of kidney allograft recipients.

Characteristics	Total	AR (n=23)	NAR (n=19)	p value
Recipient age (years)	38.2±1.7	38.5±11.3	38.3±9.2	0.067
Recipient sex (Male %)	61.9	60.9	63.2	0.879
Dialysis Vintage (Months)	11.4	32.95	7.45	0.042
	(2.75-51.95)	(5.83- 66.5)	(0-23.25)	
Induction type, n (%)				
Antithymocyte globulin	12 (28.57)	8 (34.78)	4 (21.05)	
Basiliximab	29 (69.05)	14 (60.87)	15 (78.95)	0.301
Both	1 (2.38)	1 (4.35)	0 (0)	
Kidney disease, n (%)				
GIomeruIonephritis	31 (73.81)	15 (65.22)	16 (84.21)	
Hypertension	4 (9.52)	4 (17.39)	0 (0)	0.086
Polycystic kidney disease	1 (2.38)	1 (4.35)	0 (0)	
others	6 (14.29)	3 (13.04)	3 (15.79)	
Donor age (years)	53 (41.75-58)	(38.25-57.5)	55 (46.5-59)	0.187
Donor sex (Male %)	57.1	60.9	52.6	0.591
Deceased donor(Y/N), n (%)				
				
N	26 (61.9)	10 (43.48)	16 (84.21)	
BUN (mmol/L)	18.33±0.98	19.76 ±6.64	16.32±5 .57	0.751
SCR (umol/L)	775±47	820±263	701±297	0.523
		5.85	9.7	
GFR (ml/min/1.73m2)	7.45 (5.13-10.45)	(4.68-8.35)	(6.5-12.43)	0.019
UPRO (g/L)	2.54±0 .23	2.49±1.66	2.39±1.01	0.05
UA (umol/L)	369±17	382±112	352±102	0.866

Numbers are presented as mean ± SD, median(25- 75 percentiles)or count(percentage %). AR, acute rejection; NAR, non acute rejection; CIT, cold ischemia time; BUN, blood urea nitrogen; SCR, serum creatinine; GFR, glomerular filtration rate; UPRO, urine protein; UA, Uric Acid.

3.3 RNA-Seq and DEG analysis

In our mRNA-Seq dataset, 975 genes were identified to be differentially expressed in AR compared to NAR patients based on the selection criteria of log2|FC| > 1.5 and p < 0.05 for the identification of DEGs. A total of 776 upregulated and 199 downregulated genes were identified among these 975 genes. The same method was used to identify 3084 DEGs in GES112927, 1306 DEGs in GES120396, 1205 DEGs in GES 120649, 3384 DEGs in GES 131179 and 1352 DEGs in GES 145503. The specific biological functions or pathways which these DEGs affect or are involved in are presented in the GO and pathway enrichment analyses. These genes were enriched in inflammatory and immune-related signaling pathways (**Figures 4B, C**).

**Figure 4:**
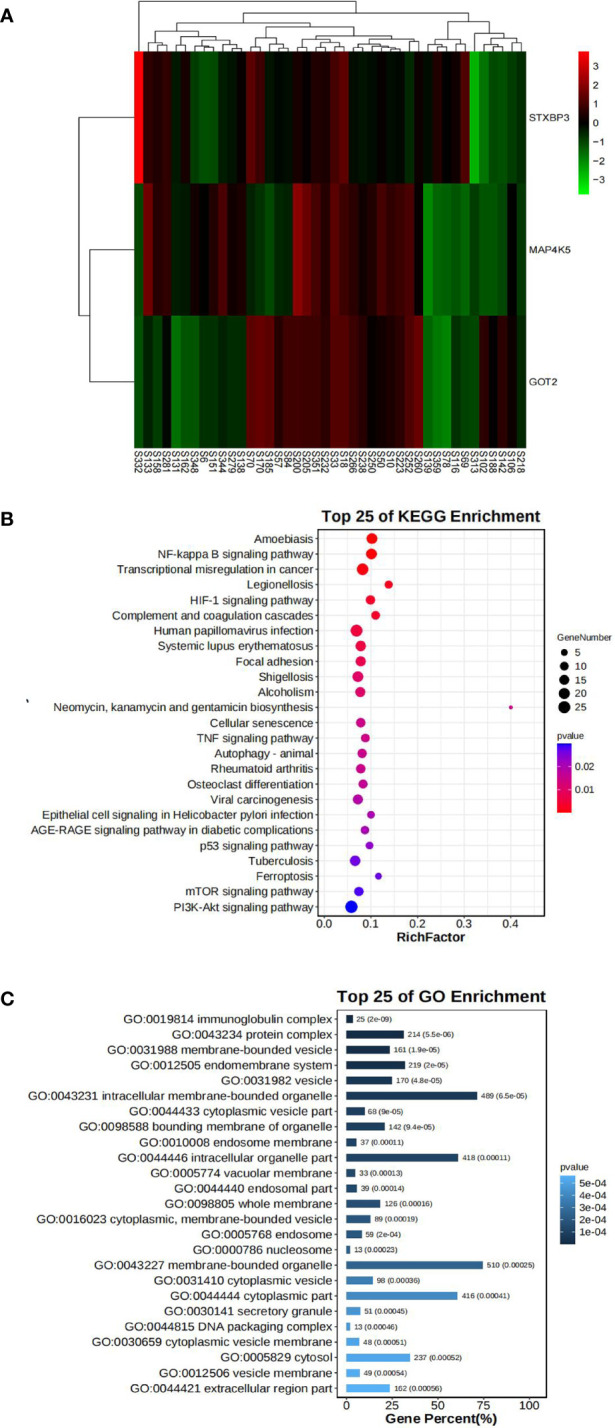
Bioinformatics analysis of our center RNA sequencing dataset. **(A)** The expression levels of STXBP3, GOT2, and MAP4K5 illustrated using a heatmap. **(B)** GO analysis of DEGs in our center dataset. **(C)** KEGG pathway analysis of DEGs in our center dataset.

3.4 Validation of key DEGs by RT-qPCR, ELISA, and IHC staining

Fifteen key DEGs were validated by collecting 14 blood samples from four patients with NAR and ten patients with AR for the RT-qPCR and observed that the mRNA levels of STXBP3, GOT2, and MAP4K5 were upregulated in the AR group compared to those in the NAR group (**Figures 5A-C**). This indicating their significant potential as biomarkers for the discrimination of AR. The expression of these three upregulated genes was visualized using a heatmap (**Figure 4A**). The serum levels of STXBP3, GOT2, and MAP4K5, were further monitored by collecting 32 blood samples from 14 patients with NAR and 18 patients with AR for ELISA. The expression of STXBP3 and GOT2 was significantly elevated in the AR group compared with that in the NAR group, while no difference in MAP4K5 levels were observed (**Figures 5D-F**). The ROC curves showed that the AUC values of STXBP3 and GOT2 were 0.980 and 0.966, respectively (**Figures 5G, H**). Finally, IHC staining wasperformed and revealed that the AR group showed significantly stronger immunocytochemical staining for both STXBP3 and GOT2 than the NAR group (**Figures 5I, J**).

**Figure 5:**
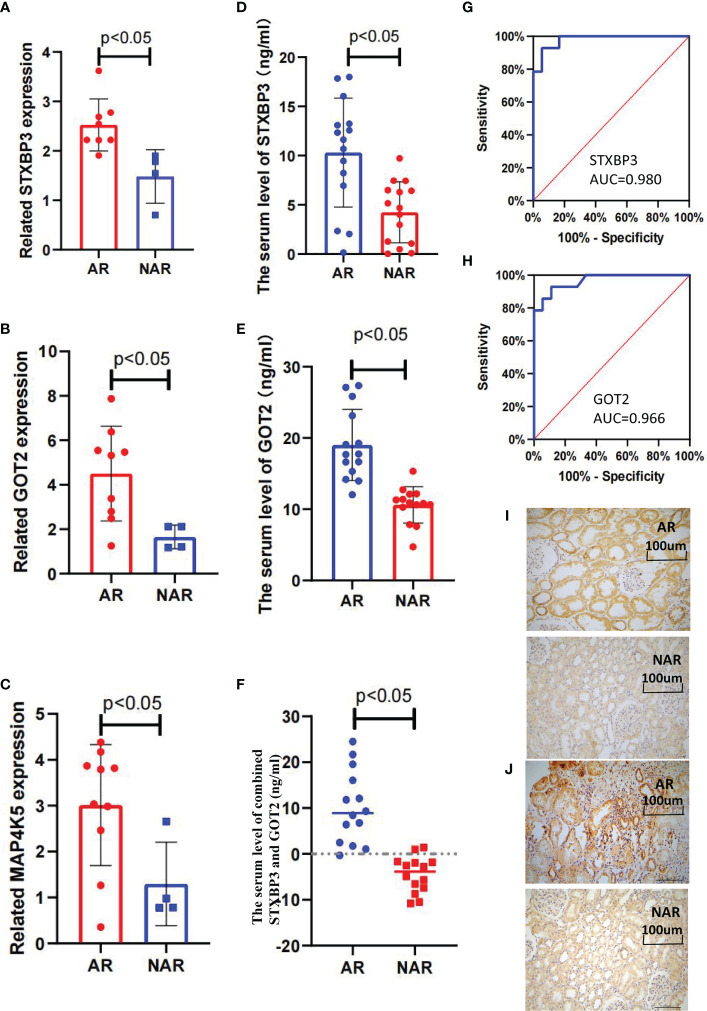
Validation of three key DEGs with RT-qPCR, ELISA, and IHC staining. **(A–C)** STXBP3, GOT2, and MAP4K5 expression was measured in 10 healthy controls, four patients with without AR episodes (NAR), and 10 patients with acute rejection (AR). **(A)** The expression of STXBP3 was compared between healthy controls, patients with NAR, and patients with AR (n = 24). **(B)** The expression of GOT2 was compared between healthy controls, patients with NAR, and patients with AR (n = 24). **(C)** The expression of MAP4K5 was compared between healthy controls, patients with NAR, and patients with AR (n = 24). **(D–F)** ELISA validation of STXBP3 and GOT2 expression in four patients with NAR and 10 patients with AR. **(G, H)** ROC curve was constructed to estimate the diagnostic power of STXBP3 and GOT2 for early AR. STXBP3: AUC = 0.989 (p < 0.0001), cut-off value = 7.840, sensitivity: 0.929, specificity: 0.944; GOT2: AUC = 0.966 (p < 0.0001), cut-off value = 13.147, sensitivity: 0.929, specificity: 0.889; and the combination of STXBP3 and GOT2: AUC = 1.000(p < 0.0001). **(I, J)** Immunohistochemical staining of kidney tissues showed that both STXBP3 and GOT2 were increased in AR group compared with that in NAR group. The scale bars in i–j = 100 μm.

4 Discussion

The occurrence of AR in kidney transplantation has been significantly reduced; however, with an AR rate of less than 10% in the first year after kidney transplantation, AR episodes still dramatically affect graft survival (14, 41–43). Therefore, prompt identification and intervention for AR are vital for improving graft outcomes (44). Here, we performed the RNA-seq to analyze the DEGs in the peripheral blood of AR recipients collected before renal transplantation and at the time of AR episode to acquire the expression changes of DEGs from pre-operation to the AR episodes.

STXBP3 and GOT2 were identified as the most significant genes for the prediction ofAR episodes and subsequently verified their higher mRNA, protein, and serum expression levels in the AR group. Moreover, the ROC curve analysis of STXBP3 and GOT2 exhibited favorable prediction performance for AR risk before kidney transplantation with a high AUC, which provides a promising opportunity to assess AR risk before recipients undergo kidney transplantation. Emerging studies indicated a correlation between STXBP3 and immune dysregulation. STXBP3 expression was found to be enriched in circulating monocytes, dendritic cells (DC), B cells, and T cells and enhanced in inflammatory fibroblasts in patients with ulcerative colitis (UC) (45). STXBP3 has also contributed to the establishment of immunological tolerance in the induction of T cell anergy by the inhibition of the calcineurin-induced calcium influx pathway and inactivation of the nuclear factor of activated T cells (NFAT) (46). GOT2 is ubiquitous in mitochondria and is associated with the regulation of cellular metabolism, especially the malate-aspartate shuttle (47–50). More importantly, GOT2 was also screened using isobaric tags for relative and absolute quantitation (iTRAQ) proteomics as a functional protein in the lupus mouse model and identified to exhibit a close association with the pathogenesis and development of lupus nephritis by reducing immune inflammation caused by active oxygen. Together, both STXBP3 and GOT2 may be involved in the regulation of immunopathogenic mechanisms (51).

The application of STXBP3 and GOT2, with two clinical indics donor age and eGFR, may facilitate the evaluation of AR risk before kidney transplantation. Monitoring the dynamics change in AR-related genes helped predict thepostoperative risks of AR episodes.

Our study has several novel aspects that offer clues for diagnosing AR early. The dynamic trends of DEGs were compared before renal transplantation and during the AR episode, dynamical monitoring of the expression changes of AR-related genes in the peripheral blood is expected to distinguishAR episodes and prompt interventions in a timely manner. The subsequent bioinformatics analysis and xCell online tool correlated gene expression profiles and functions with specific types of immune cells; therefore, critical immune cell populations of the AR process were identified, which plays a similar role to that of single-cell/single-nuclei sequencing. Additionally, the data in our study for the screening of DEGs related to AR came from multiple centers which makes these results relatively objective. However, this study had several limitations. First, we had a few samples available for clinical validation owing to the limited accessibility of the positive specimens. The precise mechanisms of STXBP3 and GOT2 have not been explored in acute renal transplant rejection. Therefore, adequate clinical cases should be further evaluated to validate the results in this study.

## Data availability statement

The original contributions presented in the study are included in the article/**Supplementary Materials**. Further inquiries can be directed to the corresponding authors.

## Ethics statement

The studies involving human participants were reviewed and approved by the Kidney Disease Center of the First Affiliated Hospital, College of Medicine, Zhejiang University. The patients/participants provided their written informed consent to participate in this study.

## Author contributions

QY performed the data curation and wrote the manuscript. WX collected the clinical data and analyzed the data. HJ designed the study and experiments. CW and YW conducted the experiments. YC and QZ interpreted the results. DC and JC supervised the manuscripts and experiments. All authors contributed to the article and approved the submitted version.

## Funding

This work was funded by NSFC81970651, U21A20350, Sino-German CENTER GZ1572, and NSF81802085.

## Conflict of interest

The authors declare that the research was conducted in the absence of any commercial or financial relationships that could be construed as a potential conflict of interest.

## Publisher’s note

All claims expressed in this article are solely those of the authors and do not necessarily represent those of their affiliated organizations, or those of the publisher, the editors and the reviewers. Any product that may be evaluated in this article, or claim that may be made by its manufacturer, is not guaranteed or endorsed by the publisher.

## Supplementary material

Supplementary Table 1 Primers of 15 genes for RT-qPCR.

Supplementary Figure 1 The flowchart of patient screening and recruitment in our study.

## References

[1] Lee Y Kwon S Moon JJ Han K Paik NJ Kim WS. The effect of health-related behaviors on disease progression and mortality in early stages of chronic kidney disease: A Korean nationwide population-based study. J Clin Med (2019) 8:1100. doi: 10.3390/jcm8081100 31349578 PMC6723181

[2] Dai Q Zhang Y Liao X Jiang Y Lv X Yuan X. Fluorofenidone alleviates renal fibrosis by inhibiting necroptosis through RIPK3/MLKL pathway. Front Pharmacol (2020) 11:534775. doi: 10.3389/fphar.2020.534775 33390935 PMC7772387

[3] Shehata M Mahmoud A Soliman A Khalifa F Ghazal M Abou El-Ghar M. 3D kidney segmentation from abdominal diffusion MRI using an appearance-guided deformable boundary. PLoS One (2018) 13:e0200082. doi: 10.1371/journal.pone.0200082 30005069 PMC6044527

[4] Knobbe TJ Douwes RM Kremer D Swarte JC Eisenga MF Gomes-Neto AW. Altered gut microbial fermentation and colonization with methanobrevibacter smithii in renal transplant recipients. J Clin Med (2020) 9:518. doi: 10.3390/jcm9020518 32075113 PMC7073595

[5] Wolfe RA Ashby VB Milford EL Ojo AO Ettenger RE Agodoa LY. Comparison of mortality in all patients on dialysis, patients on dialysis awaiting transplantation, and recipients of a first cadaveric transplant. N Engl J Med (1999) 341:1725–30. doi: 10.1056/nejm199912023412303 10580071

[6] Khalid U Newbury LJ Simpson K Jenkins RH Bowen T Bates L. A urinary microRNA panel that is an early predictive biomarker of delayed graft function following kidney transplantation. Sci Rep (2019) 9:3584. doi: 10.1038/s41598-019-38642-3 30837502 PMC6401030

[7] Lee J Kim DG Kim BS Kim MS Il Kim S Kim YS. Early hospital readmissions after ABO- or HLA- incompatible living donor kidney transplantation. Sci Rep (2019) 9:3246. doi: 10.1038/s41598-019-39841-8 30824777 PMC6397202

[8] Wang Z Han Z Tao J Wang J Liu X Zhou W. Role of endothelial-to-mesenchymal transition induced by TGF-β1 in transplant kidney interstitial fibrosis. J Cell Mol Med (2017) 21:2359–69. doi: 10.1111/jcmm.13157 PMC5618680 28374926

[9] Nian Y Xiong Z Zhan P Wang Z Xu Y Wei J. IL-21 receptor blockade shifts the follicular T cell balance and reduces *De novo* donor-specific antibody generation. Front Immunol (2021) 12:661580. doi: 10.3389/fimmu.2021.661580 33897706 PMC8064115

[10] Azad TD Donato M Heylen L Liu AB Shen-Orr SS Sweeney TE. Inflammatory macrophage-associated 3-gene signature predicts subclinical allograft injury and graft survival. JCI Insight (2018) 3:e95659. doi: 10.1172/jci.insight.95659 29367465 PMC5821209

[11] Lamb KE Lodhi S Meier-Kriesche HU. Long-term renal allograft survival in the united states: a critical reappraisal. Am J Transplant (2011) 11:450–62. doi: 10.1111/j.1600-6143.2010.03283.x 20973913

[12] Li ZH An N Huang XJ Yang C Wu HL Chen XC. Cyclosporine a blocks autophagic flux in tubular epithelial cells by impairing TFEB-mediated lysosomal function. J Cell Mol Med (2021) 25:5729–43. doi: 10.1111/jcmm.16593 PMC8184677 33949118

[13] Hart A Smith JM Skeans MA Gustafson SK Stewart DE Cherikh WS. OPTN/SRTR 2015 annual data report: Kidney. Am J Transplant (2017) 17 Suppl 1:21–116. doi: 10.1111/ajt.14124 28052609 PMC5527691

[14] Cooper JE. Evaluation and treatment of acute rejection in kidney allografts. Clin J Am Soc Nephrol (2020) 15:430–8. doi: 10.2215/cjn.11991019 PMC7057293 32066593

[15] Hart A Lentine KL Smith JM Miller JM Skeans MA Prentice M. OPTN/SRTR 2019 annual data report: Kidney. Am J Transplant (2021) 21 Suppl 2:21–137. doi: 10.1111/ajt.16502 33595191

[16] Clayton PA McDonald SP Russ GR Chadban SJ. Long-term outcomes after acute rejection in kidney transplant recipients: An ANZDATA analysis. J Am Soc Nephrol (2019) 30:1697–707. doi: 10.1681/asn.2018111101 PMC6727270 31308074

[17] Lefaucheur C Loupy A Vernerey D Duong-Van-Huyen JP Suberbielle C Anglicheau D. Antibody-mediated vascular rejection of kidney allografts: a population-based study. Lancet (2013) 381:313–9. doi: 10.1016/s0140-6736(12)61265-3 23182298

[18] Bouatou Y Viglietti D Pievani D Louis K Duong Van Huyen JP Rabant M. Response to treatment and long-term outcomes in kidney transplant recipients with acute T cell-mediated rejection. Am J Transplant (2019) 19:1972–88. doi: 10.1111/ajt.15299 30748089

[19] Halawa A. The early diagnosis of acute renal graft dysfunction: a challenge we face. the role of novel biomarkers. Ann Transplant (2011) 16:90–8. doi: 10.1155/2015/854070 21436782

[20] Wang JJ Chi NH Huang TM Connolly R Chen LW Chueh SJ. Urinary biomarkers predict advanced acute kidney injury after cardiovascular surgery. Crit Care (2018) 22:108. doi: 10.1186/s13054-018-2035-8 29699579 PMC5921971

[21] Dennen P Douglas IS Anderson R. Acute kidney injury in the intensive care unit: an update and primer for the intensivist. Crit Care Med (2010) 38:261–75. doi: 10.1097/CCM.0b013e3181bfb0b5 19829099

[22] Roedder S Sigdel T Salomonis N Hsieh S Dai H Bestard O. The kSORT assay to detect renal transplant patients at high risk for acute rejection: results of the multicenter AART study. PLoS Med (2014) 11:e1001759. doi: 10.1371/journal.pmed.1001759 25386950 PMC4227654

[23] Päivärinta J Oikonen V Räisänen-Sokolowski A Tolvanen T Löyttyniemi E Iida H. Renal vascular resistance is increased in patients with kidney transplant. BMC Nephrol (2019) 20:437. doi: 10.1186/s12882-019-1617-2 31775670 PMC6882025

[24] Arant BS Jr. Distal tubular sodium handling in human neonates: clearance studies Contrib Nephrol (1988) 67:130–7 doi: 10.1159/000415389 3061736

[25] Crowley LE Mekki M Chand S. Biomarkers and pharmacogenomics in kidney transplantation. Mol Diagn Ther (2018) 22:537–50. doi: 10.1007/s40291-018-0349-5 29971647

[26] Singh N Samant H Hawxby A Samaniego MD. Biomarkers of rejection in kidney transplantation. Curr Opin Organ Transplant (2019) 24:103–10. doi: 10.1097/mot.0000000000000606 30540576

[27] Oellerich M Shipkova M Asendorf T Walson PD Schauerte V Mettenmeyer N. Absolute quantification of donor-derived cell-free DNA as a marker of rejection and graft injury in kidney transplantation: Results from a prospective observational study. Am J Transplant (2019) 19:3087–99. doi: 10.1111/ajt.15416 PMC6899936 31062511

[28] Jaikaransingh V Kadambi PV. Donor-derived cell-free DNA (ddcf-DNA) and acute antibody-mediated rejection in kidney transplantation. Medicina (Kaunas) (2021) 57:436. doi: 10.3390/medicina57050436 34062714 PMC8147225

[29] Suhre K Schwartz JE Sharma VK Chen Q Lee JR Muthukumar T. Urine metabolite profiles predictive of human kidney allograft status. J Am Soc Nephrol (2016) 27:626–36. doi: 10.1681/asn.2015010107 PMC4731125 26047788

[30] Schaub S Nickerson P Rush D Mayr M Hess C Golian M. Urinary CXCL9 and CXCL10 levels correlate with the extent of subclinical tubulitis. Am J Transplant (2009) 9:1347–53. doi: 10.1111/j.1600-6143.2009.02645.x 19459809

[31] Hricik DE Nickerson P Formica RN Poggio ED Rush D Newell KA. Multicenter validation of urinary CXCL9 as a risk-stratifying biomarker for kidney transplant injury. Am J Transplant (2013) 13:2634–44. doi: 10.1111/ajt.12426 PMC3959786 23968332

[32] Rabant M Amrouche L Morin L Bonifay R Lebreton X Aouni L. Early low urinary CXCL9 and CXCL10 might predict immunological quiescence in clinically and histologically stable kidney recipients. Am J Transplant (2016) 16:1868–81. doi: 10.1111/ajt.13677 26694099

[33] Corey HE. Urine cytology: an underused method to diagnose acute renal allograft rejection. Pediatr Nephrol (1997) 11:226–30. doi: 10.1007/s004670050269 9090673

[34] Srinivas TR Kaplan B. Urinary biomarkers and kidney transplant rejection: fine-tuning the radar. Am J Transplant (2013) 13:2519–21. doi: 10.1111/ajt.12427 24007513

[35] Faddoul G Nadkarni GN Bridges ND Goebel J Hricik DE Formica R. Analysis of biomarkers within the initial 2 years posttransplant and 5-year kidney transplant outcomes: Results from clinical trials in organ transplantation-17. Transplantation (2018) 102:673–80. doi: 10.1097/tp.0000000000002026 PMC6018026 29189482

[36] Johnson WE Li C Rabinovic A. Adjusting batch effects in microarray expression data using empirical bayes methods. Biostatistics (2007) 8:118–27. doi: 10.1093/biostatistics/kxj037 16632515

[37] Ritchie ME Phipson B Wu D Hu Y Law CW Shi W. Limma powers differential expression analyses for RNA-sequencing and microarray studies. Nucleic Acids Res (2015) 43:e47. doi: 10.1093/nar/gkv007 25605792 PMC4402510

[38] Jia A Xu L Wang Y. Venn Diagrams in bioinformatics. Brief Bioinform (2021) 22:bbab108. doi: 10.1093/bib/bbab108 33839742

[39] Aran D Hu Z Butte AJ. xCell: digitally portraying the tissue cellular heterogeneity landscape. Genome Biol (2017) 18:220. doi: 10.1186/s13059-017-1349-1 29141660 PMC5688663

[40] Torre D Lachmann A Ma'ayan A. BioJupies: Automated generation of interactive notebooks for RNA-seq data analysis in the cloud. Cell Syst (2018) 7:556–61.e3. doi: 10.1016/j.cels.2018.10.007 30447998 PMC6265050

[41] Zhang X Han S Kang Y Guo M Hong S Liu F. SAHA, an HDAC inhibitor, synergizes with tacrolimus to prevent murine cardiac allograft rejection. Cell Mol Immunol (2012) 9:390–8. doi: 10.1038/cmi.2012.28 PMC4002334 22922441

[42] Duflot T Laurent C Soudey A Fonrose X Hamzaoui M Iacob M. Preservation of epoxyeicosatrienoic acid bioavailability prevents renal allograft dysfunction and cardiovascular alterations in kidney transplant recipients. Sci Rep (2021) 11:3739. doi: 10.1038/s41598-021-83274-1 33580125 PMC7881112

[43] Thomson AW Zahorchak AF Ezzelarab MB Butterfield LH Lakkis FG Metes DM. Prospective clinical testing of regulatory dendritic cells in organ transplantation. Front Immunol (2016) 7:15. doi: 10.3389/fimmu.2016.00015 26858719 PMC4729892

[44] Radovic T Jankovic MM Stevic R Spasojevic B Cvetkovic M Pavicevic P. Detection of impaired renal allograft function in paediatric and young adult patients using arterial spin labelling MRI (ASL-MRI). Sci Rep (2022) 12:828. doi: 10.1038/s41598-022-04794-y 35039571 PMC8764097

[45] Ouahed J Kelsen JR Spessott WA Kooshesh K Sanmillan ML Dawany N. Variants in STXBP3 are associated with very early onset inflammatory bowel disease, bilateral sensorineural hearing loss and immune dysregulation. J Crohns Colitis (2021) 15:1908–19. doi: 10.1093/ecco-jcc/jjab077 PMC8575043 33891011

[46] Macedo C Magalhães DA Tonani M Marques MC Junta CM Passos GA. Genes that code for T cell signaling proteins establish transcriptional regulatory networks during thymus ontogeny. Mol Cell Biochem (2008) 318:63–71. doi: 10.1007/s11010-008-9857-7 18597059

[47] Liu H Sun W Zhou Y Griffin N Faulkner S Wang L. iTRAQ-based quantitative proteomics analysis of sprague-dawley rats liver reveals perfluorooctanoic acid-induced lipid metabolism and urea cycle dysfunction. Toxicol Lett (2022) 357:20–32. doi: 10.1016/j.toxlet.2021.12.016 34958885

[48] Hong R Zhang W Xia X Zhang K Wang Y Wu M. Preventing BRCA1/ZBRK1 repressor complex binding to the GOT2 promoter results in accelerated aspartate biosynthesis and promotion of cell proliferation. Mol Oncol (2019) 13:959–77. doi: 10.1002/1878-0261.12466 PMC6441895 30714292

[49] Lavington E Cogni R Kuczynski C Koury S Behrman EL O'Brien KR. A small system–high-resolution study of metabolic adaptation in the central metabolic pathway to temperate climates in drosophila melanogaster. Mol Biol Evol (2014) 31:2032–41. doi: 10.1093/molbev/msu146 PMC4104311 24770333

[50] Mellis AT Misko AL Arjune S Liang Y Erdélyi K Ditrói T. The role of glutamate oxaloacetate transaminases in sulfite biosynthesis and H(2)S metabolism. Redox Biol (2021) 38:101800. doi: 10.1016/j.redox.2020.101800 33271457 PMC7711302

[51] Liao DJ Cheng XP Li N Liang KL Fan H Zhang SY. A comparative study on the incidence, aggravation, and remission of lupus nephritis based on iTRAQ technology. Comb Chem High Throughput Screen (2020) 23:649–57. doi: 10.2174/1386207323666200416151836 32297573

