# Supplementary material for: STXBP3 and GOT2 predict immunological activity in acute allograft rejection
Source: Front Immunol. 2022 Dec 1;13:1025681. doi: 10.3389/fimmu.2022.1025681 (PMC9751189; doi:10.3389/fimmu.2022.1025681)
Supplement: Supplementary Table 1 — Primers of 15 genes for RT-qPCR. [file Table_1.docx]

| Supplementary Table 1: Primers and RT-PCR conditions for 15 differentially expressed genes. | | | | |
| --- | --- | --- | --- | --- |
| Gene | Forward primer  (5'-3') | Reverse primer | Annealing temperature(℃) | PCR amplicon length(bp) |
| KIF3B  (Kinesin Family Member 3B) | TGGATGTGGATGTTAAGCTGGG | TCGGAACGTCTCATCGTACAG | 60 | 140 |
| FH  (Fumarate Hydratase) | CTCAGCACCATGTACCGAGC | TTTGGCTTGCCATTCGAGC | 60 | 151 |
| EIF4G1  (Eukaryotic Translation Initiation Factor 4 Gamma 1) | CTGTGTGACGAGCAGAAGGA | CCCAACTGTAGAAGGCATCC | 59 | 148 |
| SMARCD1  (SWI/SNF Related, Matrix Associated, Actin Dependent Regulator Of Chromatin, Subfamily D, Member 1) | TCAGTCCCGCAAGAGACCT | CCAGTTCACGAATCCTTTGAGG | 60 | 131 |
| ITGAL  (Integrin Subunit Alpha L) | AAATGGAAGGACCCTGATGCTC | TGTAGCGGATGTGTCTTTGGC | 60 | 210 |
| HNRNPUL1  (Heterogeneous Nuclear Ribonucleoprotein U Like 1) | GGCACATCAACGAGGAGGTC | CATGGCATAATGTCCGTCCG | 60 | 131 |
| MAP4K5  (Mitogen-Activated Protein Kinase Kinase Kinase Kinase 5) | GCCGTTCGTGCTTGTTAGC | GGCCTCCATCTTCACTTAGGG | 60 | 80 |
| ELP3  (Elongator Acetyltransferase Complex Subunit 3) | GCAGAAATGAGGCAGAAGCG | ATGTCTTTCCCCTGCTCGTG | 60 | 113 |
| AVIL  (Advillin) | CCTGCTTTGGAGTGGTTCTTC | GCTTGTCTTTCCAGGACTTGC | 59 | 95 |
| HNRNPL  (Heterogeneous Nuclear Ribonucleoprotein L) | TTGTGGCCCTGTCCAGAGAATT | GTTTGTGTAGTCCCAAGTATCCTG | 60 | 211 |
| PRPF19  (Pre-MRNA Processing Factor 19) | CGCCATGTCCCTAATCTGCT | TGTACTTCTCGATGAGCCGC | 59 | 104 |
| GOT2  (Glutamic-Oxaloacetic Transaminase 2) | CGTCCGCAAGTTTGTCACTG | GGCAGAAAGACATCTCGGCT | 60 | 108 |
| STXBP3  (Syntaxin Binding Protein 3) | TTCTGCGGCCAAAGTAGGTT | TATCTTCTGCCACACGACGC | 60 | 116 |
| CLIC3  (Chloride Intracellular Channel 3) | CTGTCGCCCAATAAAGGCATC | TTCAGATGTCAGGACACCCTC | 59 | 54 |
| PPM1G  (Protein Phosphatase, Mg2+/Mn2+ Dependent 1G) | GGCCTACACAGGCTTTTCCT | AATGCCAGGCTCACCAACTT | 60 | 70 |
