# Supplementary figures and images for: STXBP3 and GOT2 predict immunological activity in acute allograft rejection
Source: Front Immunol. 2022 Dec 1;13:1025681. doi: 10.3389/fimmu.2022.1025681 (PMC9751189; doi:10.3389/fimmu.2022.1025681)

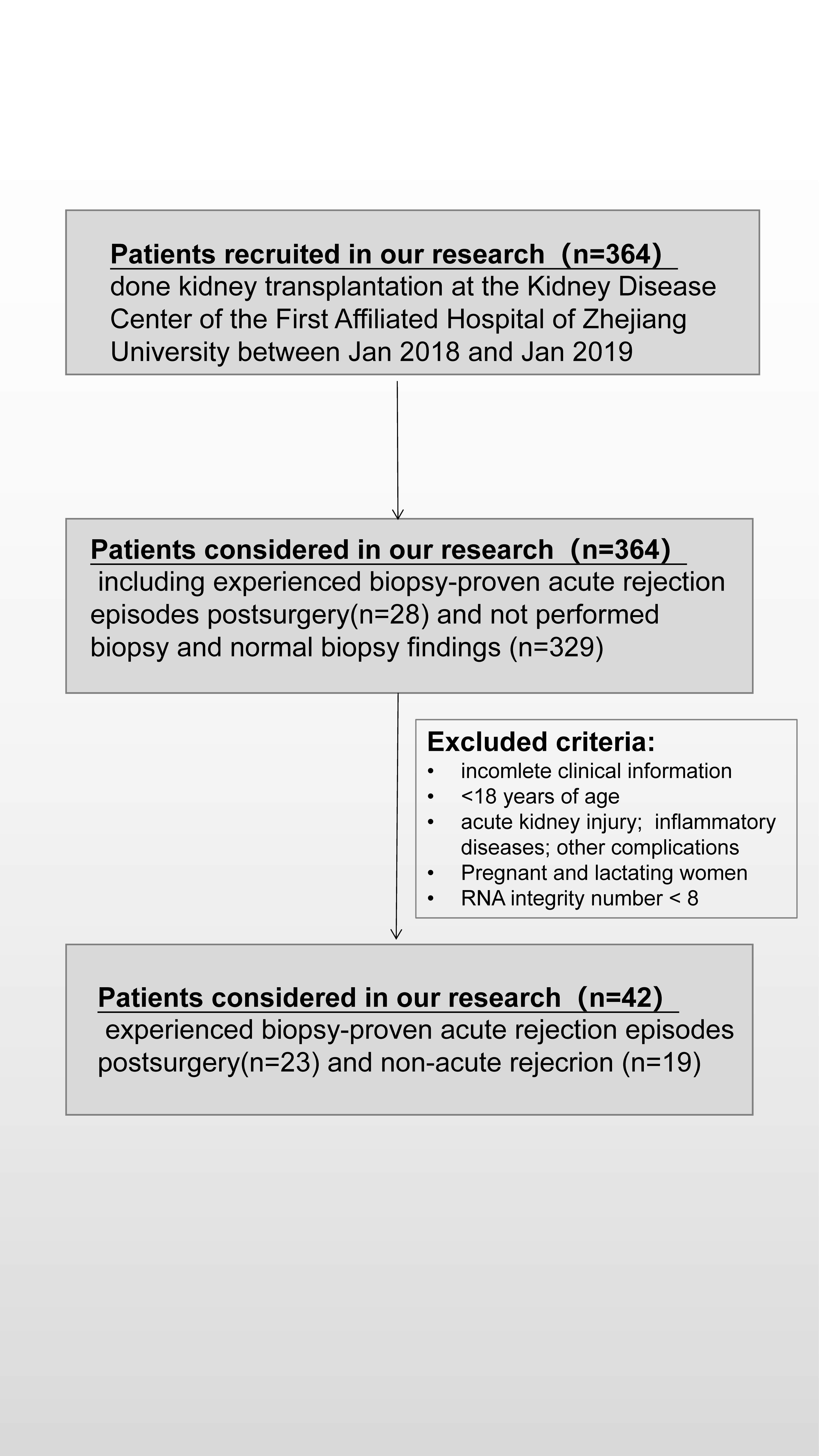

Supplement: Supplementary Figure 1 — The flowchart of patient screening and recruitment in our study. [file Image_1.tif]
